# A Piezoresistive Pressure Sensor with Optimized Positions and Thickness of Piezoresistors

**DOI:** 10.3390/mi12091095

**Published:** 2021-09-11

**Authors:** Qinggang Meng, Yulan Lu, Junbo Wang, Deyong Chen, Jian Chen

**Affiliations:** 1 Aerospace Information Research Institute, Chinese Academy of Sciences, Beijing 100190, China; mengqinggang19@mails.ucas.ac.cn (Q.M.); luyulan15@mails.ucas.ac.cn (Y.L.); chenjian@mail.ie.ac.cn (J.C.); 2 School of Electronic, Electrical and Communication Engineering, University of Chinese Academy of Sciences, Beijing 100049, China

**Keywords:** piezoresistive pressure sensor, SOI structure, low stress area, center piezoresistor, silicon connection

## Abstract

In this paper, a piezoresistive pressure sensor based on silicon on insulator (SOI) was presented, which was composed of an SOI layer with sensing elements and a glass cap for a hermetic package. Different from its conventional counterparts, the position and thickness of the four piezoresistors was optimized based on numerical simulation, which suggests that two piezoresistors at the center while the other two at the edge of the pressure-sensitive diaphragm and a thickness of 2 μm can produce the maximum sensitivity and the minimum nonlinearity. Due to the use of silicon rather than metal for electrical connections, the piezoresistive pressure sensor was fabricated in a highly simplified process. From the experimental results, the fabricated piezoresistive pressure sensor demonstrated a high sensitivity of 37.79 mV·V^−1^·MPa^−1^, a high full-scale (FS) output of 472.33 mV, a low hysteresis of 0.09% FS, a good repeatability of 0.03% FS and a good accuracy of 0.06% FS at 20 °C. A temperature coefficient of sensitivity of 0.44 mV·MPa^−1^·°C^−1^ and a low zero drift were also shown at different temperatures. The piezoresistive pressure sensor developed in this study may function as an enabling tool in pressure measurements.

## 1. Introduction

Since C.S. Smith discovered the piezoresistive effect in Si and Ge in 1954 [1], theoretical analysis and practical applications of piezoresistive sensors have made great progress. As an important branch of piezoresistive sensors, piezoresistive pressure sensors are characterized by simple fabrication and low cost in pressure measuring devices, which are widely used in numerous fields, such as medical diagnostics, industrial control and vehicle engineering [2,3,4,5].

Initially, piezoresistive pressure sensors were fabricated by partial implants of boron ions into n-type silicon diaphragms to form piezoresistors [6,7,8]. However, they suffered from compromised performances at high temperatures due to leakage currents of PN junctions for electrical isolations and complex fabrication processes requiring multi-step ion implantation and thermal annealing [9,10,11].

In order to address this issue, silicon on insulators (SOI) with a uniform diffusion of boron or phosphorus ions on the device layer were patterned to form piezoresistive pressure sensors, which were featured with low leakage currents and simplified fabrication processes [12,13,14,15,16,17,18]. More specifically, in 2005, Y.L. Zhao et al. developed a piezoresistive pressure sensor based on the SIMOX technology where four piezoresistors were positioned at the edge of a circle diaphragm, reporting a full-scale output of 95.5 mV under the current excitation of 5 mA [15]. However, the relatively low full-scale outputs, which represent comprehension performances of piezoresistive pressure sensors such as sensitivities and resolutions, made the sensor prone to environmental interferences. In 2019, Sheeparamatti B.G. put forward a piezoresistive pressure sensor with a square diaphragm based on polysilicon on insulator to increase the output voltage to 147 mV under the voltage excitation of 10 V [13]. Meanwhile, the limited full-scale outputs result in relative lower resolutions in pressure sensing. In order to improve the full-scale outputs of the piezoresistive pressure sensor, Chuang Li fabricated a four-grooved diaphragm combined with a roof beam in 2020, which was able to concentrate the stresses and gained a high full-scale output of 154.5 mV with 5 V voltage excitation [19]. Nonetheless, this is still not enough in high-precision sensing.

Various methods were used to improve the sensitivity and maximum output voltage of the sensor, such as using nanomaterials with high piezoresistive effect to make the piezoresistors [20,21,22], making the beam structure on the surface of the diaphragm for stress concentration [19], and making an island structure on the back of the diaphragm to simultaneously increase the sensitivity and decrease the nonlinearity [23]. However, detailed analysis of the stress distribution inside the sensing structures with a certain thickness was missing, leading to the failure of the assumption that the sensing structures have the same stress as the pressure-sensitive diaphragm, resulting in incomplete analysis and compromised device performance.

In order to address this issue, based on numerical simulations, this study conducted a comprehensive analysis of stress distributions inside the piezoresistors and then positioned two central and two side piezoresistors on the diaphragm and set the thickness of piezoresistors to 2 μm, leading to higher sensitivity and linearity without any changes in the materials and structures. In addition, silicon rather than metal connections were used in this study, which significantly simplified the fabrication process and minimized manufacturing errors. The piezoresistive pressure sensor developed in this study may function as an enabling tool in pressure measurements.

## 2. Structure, Theory and Simulation

### 2.1. Sensor Structure

**Figure 1:**
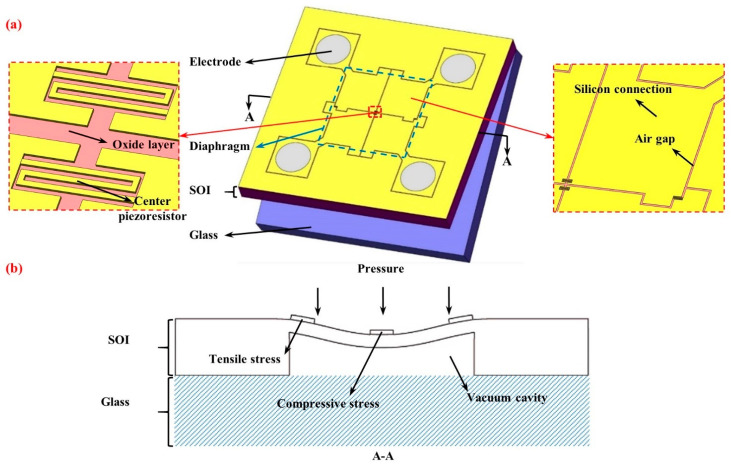
Schematic of the developed piezoresistive pressure sensor with optimized positions of piezoresistors: (**a**) The piezoresistive pressure sensor comprises two sections: an SOI wafer with sensing elements and a glass cap for a hermetic package; (**b**) outside pressure deforms pressure-sensitive diaphragms, leading to the different stresses and corresponding resistance changes of the piezoresistors.

The structure of the developed piezoresistive pressure sensor with a size of 5 mm × 5 mm × 0.9 mm is shown in Figure 1a, and was composed of an SOI wafer ((1 0 0) plane, p-type with a doping concentration of 4.5 × 10^18^ cm^−3^) with sensing elements and a glass wafer (500 μm) for a hermetic package. As shown in Figure 1a, on the device layer (2 μm thickness) of the SOI wafer, two snake-shaped piezoresistors (4 μm width and 250 μm length in total, <1 1 0> direction) of the sensor were positioned at the center, while the other two were positioned at the edge of the pressure-sensitive diaphragm (2 mm × 2 mm × 95 μm), which was formed by etching a vacuum cavity on the handle layer (400 μm thickness) of the SOI wafer. Then, four piezoresistors formed a Wheatstone bridge based on electrical connections of silicon derived from the device layer, which were isolated from each other by air gaps in this layer and isolated from the handle layer by the oxide layer (1 μm thickness). In addition, four circular Al electrodes (1 μm thickness) were positioned at the corners to conduct electrical signals.

Figure 1b shows the cross-section of the diaphragm and the working principle of the developed piezoresistive pressure sensor. Facing the difference between the outside pressure and the vacuum cavity formed by the pressure-sensitive diaphragm and the glass cap, the diaphragm and the piezoresistors positioned on the diaphragm experience corresponding deformations. Then, the piezoresistors undergo a variety of stresses and produce different resistance changes, which are converted to voltage output by the Wheatstone bridge.

### 2.2. Fundamental Theory

The piezoresistive coefficients $\pi_{11}$, $\pi_{12}$ and $\pi_{44}$, which are the key parameters of piezoresistive pressure sensors and play the role of converting the stresses in silicon into corresponding resistance changes, vary with doping concentration, doping type and crystal orientation [24]. For the most commonly used p-type silicon wafers with (1 0 0) orientation, $\pi_{11}$ and $\pi_{12}$ are significantly smaller than $\pi_{44}$, and can be ignored. In addition, the gauge factor (G=πE) achieves the maximum value in the <1 1 0> direction, which is the optimal piezoresistor orientation. Thus, for the [1 1 0]-oriented piezoresistors, the relative change in the resistance as a function of stress is shown in Equation (1).

(1) $$\frac{△R}{R}=\pi_{l}\sigma_{l}+\pi_{t}\sigma_{t}=\frac{\pi_{11}+\pi_{12}+\pi_{44}}{2}\sigma_{l}+\frac{\pi_{11}+\pi_{12}-\pi_{44}}{2}\sigma_{t}\approx \frac{\pi_{44}}{2}\left(\sigma_{l}-\sigma_{t}\right)$$

where R and △R denote the resistance of piezoresistors and the change in resistance, respectively, $\pi_{l}$ and $\pi_{t}$ are transverse and longitudinal piezoresistive coefficients, respectively, $\sigma_{l}$ is the longitudinal stress in the piezoresistors and $\sigma_{t}$ is the transverse stress in the piezoresistors.

Based on the assumption that four piezoresistors in the Wheatstone bridge have the same resistances but different resistance changes ($R_{1}=\;R_{2}=R_{3\;}=R_{4},\;\;△R_{1}=△R_{3}\neq △R_{2}=△R_{4}$), the output voltage of the Wheatstone bridge can be described by Equation (2):

(2) $$U_{O}=\frac{1}{2}U_{I}\left(\frac{△R_{1}}{R_{1}}-\frac{△R_{2}}{R_{1}}\right)$$

Substitute Equation (1) into Equation (2):

(3) $$\begin{array}{c} U_{O}=\frac{1}{4}U_{I}\pi_{44}\left(\left(\sigma_{l1}-\sigma_{t1}\right)-\left(\sigma_{l2}-\sigma_{t2}\right)\right)\\ =\frac{1}{4}U_{I}\pi_{44}\left(\Delta \sigma_{1}-\Delta \sigma_{2}\right)\\ =\frac{1}{4}U_{I}\pi_{44}\Delta \sigma_{R} \end{array}$$

where $\Delta \sigma_{1}$ and $\Delta \sigma_{2}$ denote the difference between longitudinal stress and transverse stress in different piezoresistors ($\Delta \sigma_{1\;}=\sigma_{l1}-\sigma_{t1}$,$\Delta \sigma_{2}=\;\sigma_{l2}-\sigma_{t2}$). $\Delta \sigma_{R}$ denotes the difference between $\Delta \sigma_{1}$ and $\Delta \sigma_{2}$ ($\Delta \sigma_{R}=\Delta \sigma_{1}-\Delta \sigma_{2}$). For simplicity, we name $\Delta \sigma_{1}$ and $\Delta \sigma_{2}$ as absolute stress difference (ASD), which is proportional to the resistance change of an individual piezoresistor. $\Delta \sigma_{R}$ is called the relative stress difference (RSD), which is proportional to the output of the Wheatstone bridge. Hence, the positions and parameters of piezoresistors should be optimized to obtain the maximum ASD and the corresponding maximum resistance change.

### 2.3. Numerical Simulation

Finite element analysis was used in the design and optimization of the piezoresistive pressure sensor to mainly address three problems: the position, the thickness of the piezoresistors and the material of the electrical connections. Appropriate positions and thickness should be found to obtain the maximum stresses inside the piezoresistors to produce the maximum resistance changes. The materials of electrical connections may affect the output of the Wheatstone bridge, which also deserved detailed analysis.

**Figure 2:**
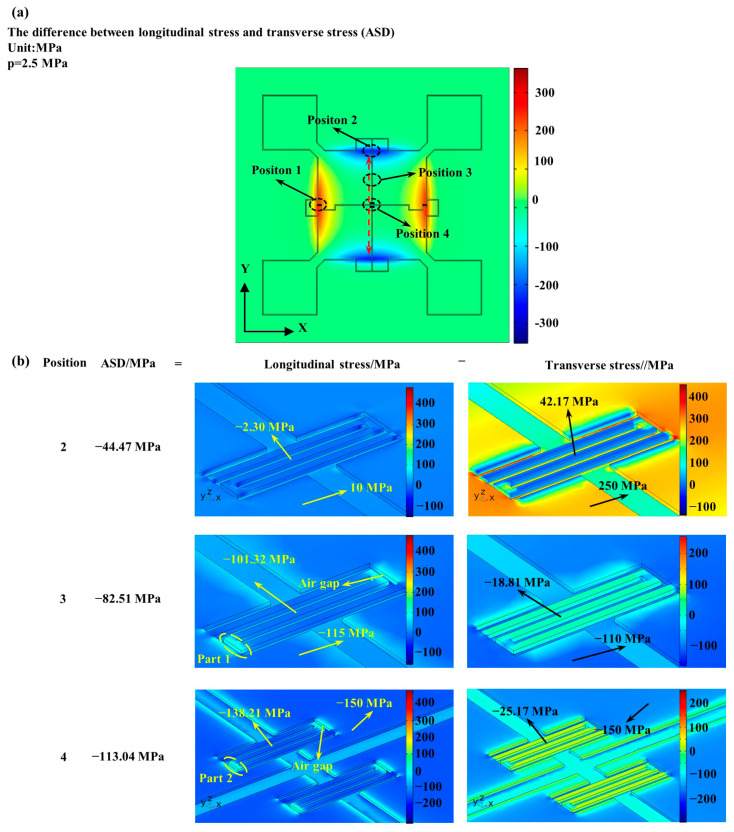
Numerical simulation of the surface stresses of the developed piezoresistive pressure sensor: (**a**) the absolute stress difference (ASD) of the proposed sensor with (**b**) the detailed distribution of longitudinal/transverse stresses inside the piezoresistors at positions 2, 3 and 4.

The sensor was simulated using the COMSOL Multiphysics (5.5 version, COMSOL Inc., Stockholm, Sweden). Figure 2a shows the simulation model of the piezoresistive pressure sensor meshed with 304,076 hexahedral elements. In this study, the field of solid mechanics was used for stress analysis, where a pressure of 2.5 MPa was applied to the diaphragm as the load and a fixed constraint was applied to the loop box at the bottom of SOI as the boundary condition. Furthermore, the field of electric currents was used to apply an electrical excitation of 5 V DC to the Wheatstone bridge with the corresponding voltage output measured. In the end, the field of multiphysics was used to couple the fields of solid mechanics and electric currents and formed a connection between stress variation and resistance changes based on the piezoresistive effects.

Figure 2a shows the distribution of the absolute stress difference (ASD), which was defined as the difference between longitudinal (X-axis) and transverse (Y-axis) stresses and was proportional to the resistance changes of piezoresistors, as shown in Equation (1) [25]. For two piezoresistors with 2 μm thickness positioned at the edge of the pressure-sensitive diaphragm in X-direction (position 1), a volume-averaged ASD was calculated as 221.56 MPa under the pressure of 2.5 MPa. As for the other two piezoresistors with same thickness moved in the Y-direction along the pressure-sensitive diaphragm with different distances to the center of the diaphragm, the values of ASD were quantified as −44.47 MPa for position 2 with a distance of 1000 μm, −82.51 MPa for position 3 with a distance of 421 μm, and −113.04 MPa for position 4 with a distance of 35 μm, which showed a decreasing trend as the distance increased.

Figure 2b shows the detailed longitudinal stresses and transverse stresses of piezoresistors at positions 2, 3 and 4, with the purpose being to analyze the stress distribution on the surface of piezoresistors. For the longitudinal stresses of three positions, several low-stress areas (LSA) existed at the edge of piezoresistors (part 1 and part 2) due to the air gaps for electrical isolation, which had limited areas and just occupied a small part of piezoresistors. Due to the influence of LSA, the absolute value of longitudinal stresses was slightly reduced compared with the diaphragm region around them (e.g., piezoresistors at position 3 showed lower longitudinal stress of −101.32 MPa compare with −115 MPa on the diaphragm). For the transverse stresses of three positions, the LSA also existed in the piezoresistors but occupied almost the whole volume because of the short width of piezoresistors compared with the length. Due to that, the absolute value of transverse stresses was greatly reduced to close to 0. Hence, the trend of decreasing ASD with increasing distance of two piezoresistors were dominated by the corresponding longitudinal stresses, which were −2.30, −101.32 and −138.21 MPa for positions 2, 3 and 4, respectively, while the contributions of the transverse stresses were negligible, at 42.17, −18.81 and −25.17 MPa for positions 2, 3 and 4, respectively, due to the existence of air gaps acting as stress isolation structures.

**Figure 3:**
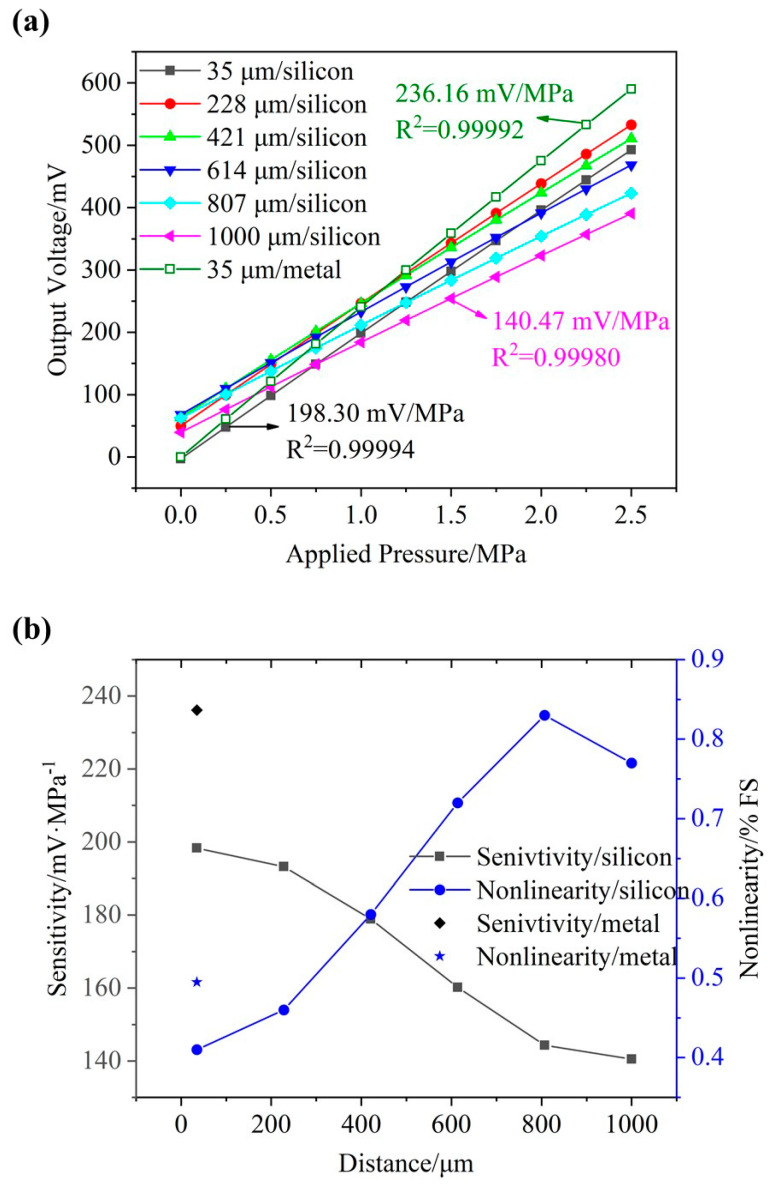
(**a**) The voltage output of the piezoresistive pressure sensor as a function of the applied pressure when the piezoresistors on the Y-centerline of the pressure-sensitive diaphragm were positioned at different distances from the center, with corresponding sensitivities and linearities (**b**).

Figure 3a shows the voltage output of the piezoresistive pressure sensor as a function of the applied pressure when the piezoresistors on the Y-centerline of the pressure-sensitive diaphragm were positioned at different distances from the center. As the distance between two piezoresistors was increased from approximately 35 to 1000 μm with an interval of 193 μm, zero drifts were observed to increase from −2.9 to 68.1 mV because of the increased mismatch of the Wheatstone bridge.

Figure 3b shows the sensitivity and linearity of the piezoresistive pressure sensor as a function of the applied pressure when the piezoresistors on the Y-centerline of the pressure-sensitive diaphragm were positioned at different distances from the center. More specifically, as the distance between two piezoresistors was increased from 35 to 1000 μm, output sensitivities were found to decrease from 198.30 to 140.47 mV/MPa, and nonlinearities were observed to increase from 0.41% FS to 0.77% FS. These results were consistent with the results showed in Figure 2b, which further indicated two piezoresistors should be positioned at the center of the pressure-sensitive diaphragm.

**Figure 4:**
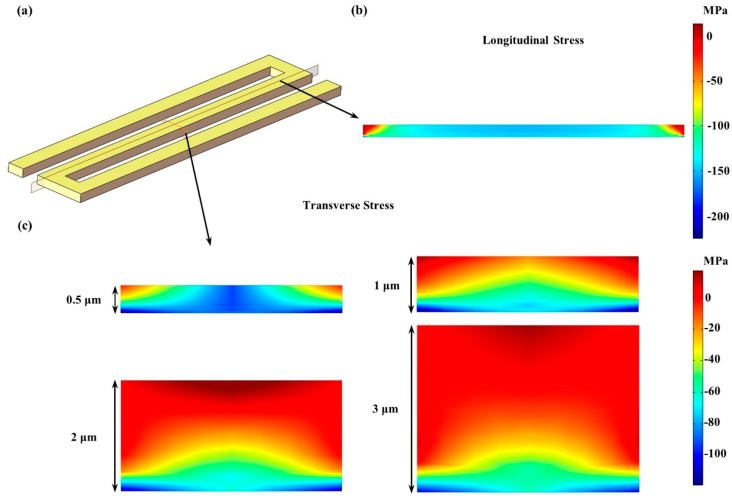
Numerical simulation of the inside stresses of the developed piezoresistive pressure sensor: (**a**) the piezoresistor model at the center of the diaphragm with two cross sections in different directions; (**b**) the longitudinal stress on the longitudinal section when the thickness of piezoresistor was 3 μm; (**c**) the transverse stress on the transverse section when the thickness of piezoresistor varies from 0.5 to 3 μm.

To further analyze the stress distribution and find the optimum thickness of piezoresistors, detailed analyses of longitudinal/transverse stresses were conducted inside the center piezoresistor, as shown in Figure 4a, with the corresponding longitudinal section and the transverse section appropriately marked.

Figure 4b shows the longitudinal stress on the longitudinal section when the thickness of piezoresistor was 3 μm. Uneven stress distribution was presented inside the piezoresistor with two LSA (the red region) at the edge and relatively uniform stress in the middle. The LSA presented an inverted triangle shape and occupied a certain area that increased with thickness, which decreased the absolute value of the average longitudinal stress of the piezoresistor.

Figure 4c shows the transverse stress on the transverse section when the thickness of piezoresistor varies from 0.5 to 3 μm. The stress distribution was similar to Figure 4b, with two small and separate LSA at the edge when the thickness was 0.5 μm but an enlarged and connected LSA at the top when the thickness increased to 1 μm. As the thickness continued to increase to 2 and 3 μm, LSA gradually expanded and occupied most of the volume of the piezoresistor under the premise that the stress distribution at the bottom did not fundamentally change, which led the average transverse stress of the piezoresistor to be closer to 0.

**Figure 5:**
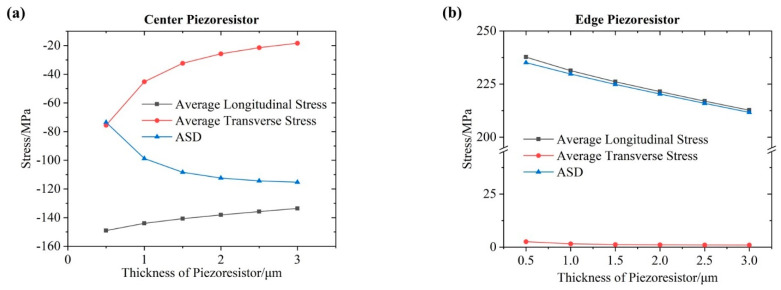

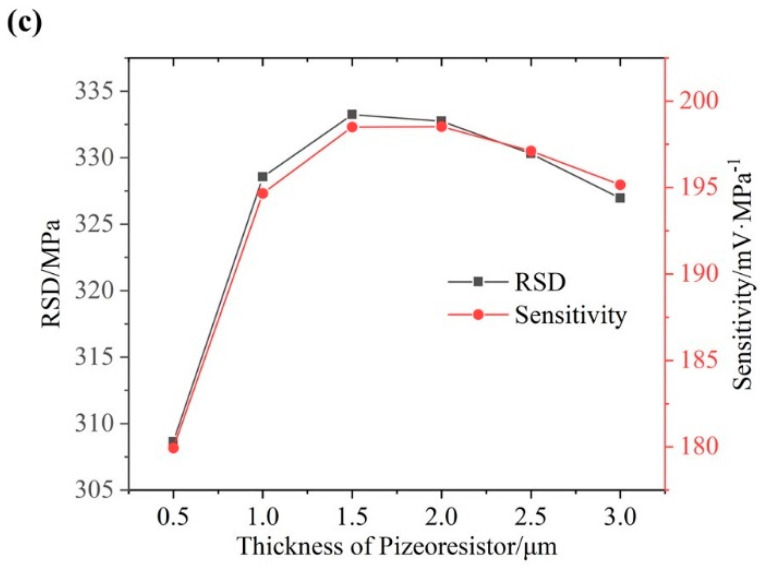
(**a**) The average longitudinal/transverse stresses and ASD of the center piezoresistor as a function of the thickness; (**b**) the average longitudinal/transverse stresses and ASD of the edge piezoresistor as a function of the thickness; (**c**) the RSD between the center piezoresistor and the edge piezoresistor and the sensitivity of the developed sensor as a function of the thickness.

Figure 5a shows the average longitudinal/transverse stresses and ASD of the center piezoresistor as a function of the thickness. The longitudinal stress, which has two relatively small LSA at the edge, would increase approximately linearly with the expansion of LSA. On the contrary, the transverse stress, which was mostly occupied by a large LSA, showed a lower increasing trend as the thickness increased. The different ascent rates led to a decreasing trend of ASD, which meant that the center resistance change under certain pressures would increase with the increasing thickness of piezoresistor (the absolute value of ASD increased).

Figure 5b shows the average longitudinal/transverse stresses and ASD of the edge piezoresistor as a function of the thickness. Similarly to the observations discussed above, the longitudinal and transverse stresses showed the same decreasing trend but different descent rates as the thickness increased, leading to a decreasing trend of ASD, which meant the edge resistance change under a certain pressure would decrease with the increasing thickness of the piezoresistor.

Figure 5c shows the relative stress difference (RSD) as a function of the thickness, which was defined as the difference between the ASD of center/edge piezoresistors, corresponding with the sensitivity of the developed sensor. The RSD and the sensitivity first rose and then fell with the biggest value at 1.5 or 2 μm, due to the different descent rates between the ASD of the center/edge piezoresistors. Considering the resistivity of piezoresistors, a thickness of 2 μm was chosen to obtain the maximum stress inside the piezoresistors and thus the highest pressure sensitivity of the proposed sensor.

Furthermore, output voltages as a function of the applied pressure based on silicon and metal connections when 2 μm-thickness piezoresistors were placed at the center are shown in Figure 3a and b for comparison. Compared with silicon-based electrical connection with a sensitivity of 198.30 mV/MPa and a nonlinearity of 0.41% FS, the metal-based electrical connection produced a limited increase of 236.16 mV/MPa for sensitivity, but a worse nonlinearity of 0.50% FS. The increase in sensitivity was caused by the lower resistance of metal which would not share the input voltage compared with silicon. The increase in nonlinearity was caused by the unbalance of the Wheatstone bridge, which was well-balanced considering the resistance of the silicon connections in the design. Since the utilization of silicon as the material of electrical connection can greatly simplify the manufacturing processes and eliminate the residual stress in metal deposition, silicon rather than metal was used in this study.

## 3. Fabrication

**Figure 6:**
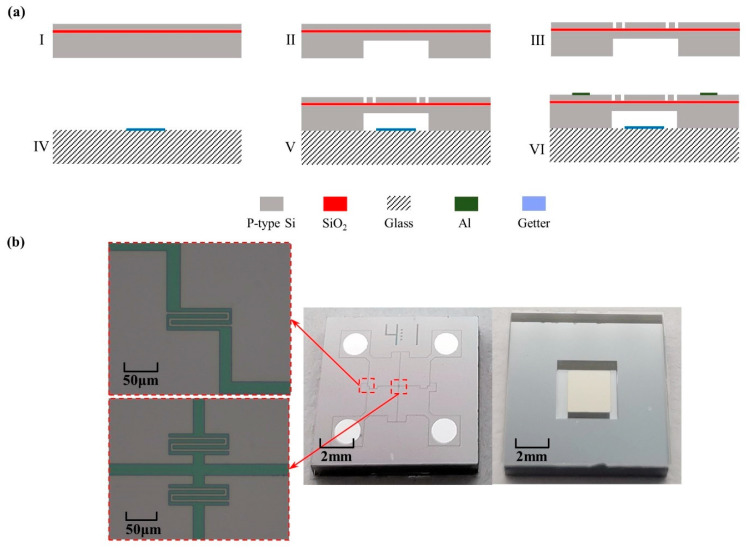
Fabrication of the developed piezoresistive pressure sensor with optimized positions of piezoresistors: (**a**) Fabrication processes include key steps of: (I) cleaning the SOI wafer, (II) etching the handle layer to form the vacuum cavity, (III) etching the device layer to form the Wheatstone bridge, (IV) evaporating the getter, (V) anodic bonding and (VI) evaporating Al electrodes; (**b**) The front and back views of the fabricated sensor with the details of piezoresistors.

A 4″ SOI wafer and a 4″ BF33 glass wafer were used in device fabrication as follows (see Figure 6a).

The SOI wafer was cleaned using piranha etchant (H_2_SO_4_:H_2_O_2_ = 5:1) to remove organic impurities (see Figure 6a(I)). Then, the vacuum cavity in the handle layer and the piezoresistors in the device layer of the SOI wafer were etched by deep reactive ion etching (DRIE) based on patterned AZ4903 and AZ1500 photoresist, respectively (see Figure 6a(II), (III)). Films of Ti with a thickness of 1 μm and Au with a thickness of 30 nm were evaporated on the BF33 glass wafer successively as getter materials (see Figure 6a(IV)), followed by anodic bonding to form the vacuum cavity (see Figure 6a(V)). In the end, four Al electrodes with a thickness of 1 μm were evaporated on the patterned device layer to form electrical connections (see Figure 6a(VI)).

Figure 6b shows the fabricated piezoresistive pressure sensor with a size of 5 mm × 5 mm × 0.9 mm as well as piezoresistors at the edge and center of the pressure-sensitive diaphragm, respectively.

Then, Kovar metal was used as the packing metal because of its high reliability, stability and low coefficient of thermal expansion (6 × 10^−6^/°C). Firstly, the adhesive was coated to the bottom of the sensor at room temperature. Secondly, the sensor chip was positioned on the Kovar base and allowed to rest for a few days to release the stress. Then, the electrodes of the sensor and the pins of the Kovar base were connected by a golden wire. Finally, the cylindrical shell was bonded to the Kovar base by soldering.

## 4. Characterization

**Figure 7:**
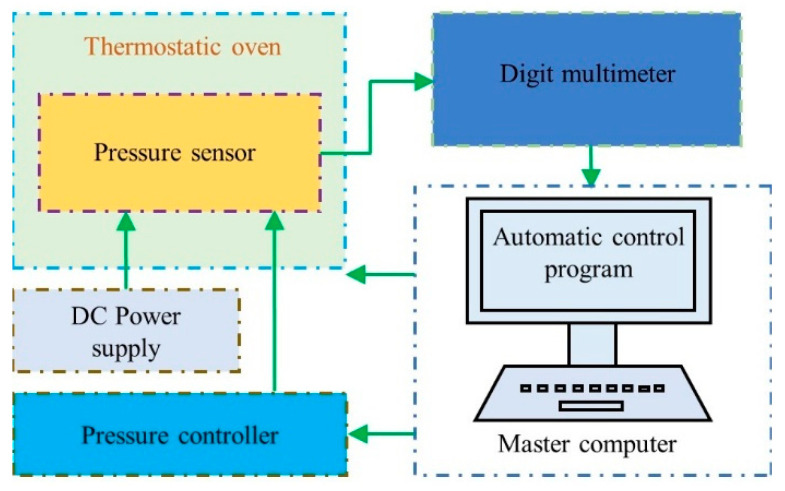
Sensor characterization system.

The characterization system of the fabricated piezoresistive pressure sensor mainly included a pressure controller (PPC4, DH Instruments, Everett, WA, USA, accuracy of 10 Pa), a thermostatic oven (SH 241, ESPEC, Osaka, Japan, accuracy of 0.5 °C) and a digit multimeter (KEITHLEY 2100, Tektronix, Beaverton, OR, USA, accuracy of 0.0038%), as shown in Figure 7.

In characterization, input parameters included pressure with a range of 0.25 to 2.5 MPa and temperature with a range of −40 to 60 °C due to the limitation of thermostatic oven. The output parameters were the voltage of the Wheatstone bridge under the 5 V excitation voltage or the calculated pressure obtained by output voltage.

**Figure 8:**
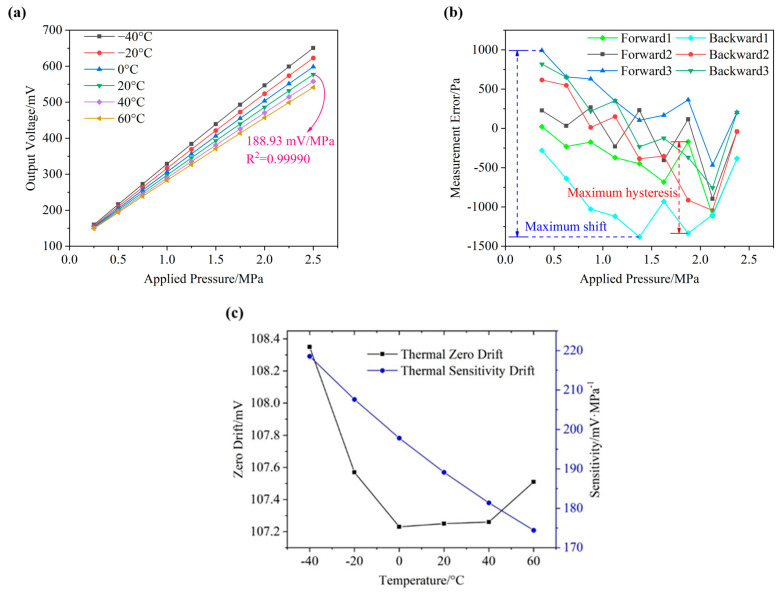
Characterization of the developed piezoresistive pressure sensor with optimized positions of piezoresistors: (**a**) the output voltage under the functions of pressure and temperature variations; (**b**) the measurement errors as a function of cycled pressure at a temperature of 20 °C; (**c**) the zero drift and sensitivity drift as a function of temperature from −40 to 60 °C.

Figure 8a shows the output voltage of the fabricated piezoresistive pressure sensor under the functions of pressure and temperature variations. At a certain temperature, a linear correlation between the output voltage and applied pressure was found, which had a sensitivity of 218.18 mV/MPa and a R2 value (correlation coefficient) of 0.99979 at −40 °C, a sensitivity of 188.93 mV/MPa and a R2 value of 0.99990 at 20 °C and a sensitivity of 174.22 mV/MPa and a R2 value of 0.99993 at 60 °C.

Figure 8b shows the measurement errors as a function of cycled pressure under the temperature of 20 °C, which were obtained by curve fitting to the output voltages. The original cycled output voltages were shown in Table 1. The maximum shift of the fabricated piezoresistive pressure sensor in three pressure cycles was recorded as 2374 Pa, corresponding to 0.09% of the full-scale output, and the maximum hysteresis shift was quantified as 1167 Pa, corresponding to 0.04% of the full-scale output. Then, a hysteresis of 0.09% FS, a repeatability of 0.03% FS and an accuracy of 0.06% FS were obtained from the cycled results.

Figure 8c shows the zero voltage drift and sensitivity drift as a function of temperature from −40 to 60 °C. As the temperature increased, the sensitivity was decreased from 218. to 174.4 mV/MPa, because the piezoresistive coefficient decreased with the increasing temperature. The zero drift of the fabricated sensor changed little at different temperatures, which indicates that the Wheatstone bridge was well-balanced and would not be affected by the thermal resistance changes.

Table 2 shows the comparison results of performance with other sensors, which shows the extremely high full-scale output and good linearity of the fabricated sensor.

## 5. Conclusions

This study demonstrates the piezoresistive pressure sensor with two piezoresistors positioned at the center while the other two are positioned at the edge of the pressure-sensitive diaphragm. Based on the simulation, longitudinal stress rather than transverse stress in piezoresistors deserves more consideration due to the stress loss caused by thickness, especially when the piezoresistors have a large aspect ratio and thickness. Therefore, for an SOI wafer with a non-negligible thickness of the device layer, the piezoresistors should be designed at the extreme value area of the longitudinal stress of the pressure-sensitive diaphragm. In addition, the thickness of the piezoresistors should also be taken into consideration to weigh the stress loss of piezoresistors at different positions, which is missing in the state-of-the-art. The developed piezoresistive pressure sensor was successively fabricated based on well-established SOI MEMS. Experimental characterization confirmed the high sensitivity (full-scale output of 472.3 mV) and linearity (R2 value of 0.99990) of the fabricated piezoresistive pressure sensors because of the optimal position and thickness of the piezoresistors.

## Figures and Tables

**Table 1:** Output of the sensor under the cycle test.

Input/MPa	Output	Forward1	Backward1	Forward2	Backward2	Forward3	Backward3
0.375	Voltage/mV	176.979	177.038	176.939	176.863	176.790	176.824
0.625		225.613	225.692	225.562	225.462	225.441	225.442
0.875		273.975	274.140	273.890	273.939	273.820	273.899
1.125		322.085	322.228	322.058	321.985	321.947	321.946
1.375		369.787	369.964	369.658	369.775	369.682	369.746
1.625		417.057	417.103	417.005	416.995	416.897	416.952
1.875		463.657	463.874	463.604	463.795	463.558	463.694
2.125		509.935	509.933	509.896	509.923	509.817	509.870
2.375		555.264	555.264	555.202	555.202	555.158	555.158

**Table 2:** Comparison of sensor performance.

Sensor	This article	Sheeparamatti, B.G. [13]	Zhao, Y.L. [15]	Li, C. [19]
Structure	SOI	PolySOI	SOI	SOI
Power supply	5 V	10 V	5 mA	5 V
Pressure range	0~2.5 MPa	0~1 MPa	0~25 MPa	0~1 psi
Temperature range	−40 °C~60 °C	0 °C~400 °C	0 °C~200 °C	−25 °C~150 °C
Full-scale output	472.3 mV	147 mV	95.5 mV	154.5 mV
R2 value of the output	0.99990	0.99945	/	/
